# Are Cytomorphogenetic Events Correlated with Oral Mucosal Lesions Induced by Crack Cocaine Use? A Systematic Review

**DOI:** 10.3390/pathophysiology30040045

**Published:** 2023-12-05

**Authors:** Thiago Guedes Pinto, Milena de Barros Viana, Patricia Ramos Cury, Manoela Domingues Martins, Jean Nunes dos Santos, Daniel Araki Ribeiro

**Affiliations:** 1 Department of Biosciences, Institute of Health and Society, Federal University of São Paulo, Santos 11050-020, SP, Brazil; guedes.pinto@unifesp.br (T.G.P.); milena.viana@unifesp.br (M.d.B.V.); 2 Department of Dental Clinics, School of Dentistry, Federal University of Bahia, Salvador 40170-110, BA, Brazil; patcury@yahoo.com (P.R.C.); jeanpatol@gmail.com (J.N.d.S.); 3 Department of Pathology and Stomatology, School of Dentistry, Federal Universty of Rio Grande do Sul, Porto Alegre 90010-150, RS, Brazil; manomartins@gmail.com

**Keywords:** crack cocaine, DNA damage, genotoxicity, oral mucosal lesions

## Abstract

The aim of this systematic review was to answer the question of whether crack cocaine can induce cellular and molecular alterations and whether such alterations are somehow related to clinical lesions in the oral mucosa. The searches were undertaken in three electronic databases and conducted based on the PRISMA 2020 statement. Eleven studies published between 1994 and 2020 were analyzed. The quality of the included studies was assessed by two independent reviewers (TGP and DAR) through a confounder’s categorization methodology, in which final ratings were attributed (strong, moderate or weak) for each study. From 11 studies included, 7 evaluated the cellular/molecular impact of the addiction in a total of 492 individuals and compared to a control (non-exposure) group (n = 472). The main tests used for cellular alteration were MN and AgNORs. Cells from crack cocaine groups exhibited increased proliferation and MN counting. Only four studies evaluated the prevalence of oral lesions. All of them showed that individuals exposed to crack cocaine presented an increased number of oral lesions. Most studies showed good quality. In conclusion, our results demonstrate that crack use may induce changes at the cellular and molecular level and also exhibit an increased number of oral lesions. However, a correlation between such changes and oral mucosa lesions still needs further investigation and elucidation through other clinical studies in humans.

## 1. Introduction

It has been established that 243 million people between 15 and 64 years old use some form of illicit drug around the world, which adds up to approximately 5000 deaths due to drugs every year in Latin America and the Caribbean [1]. Data from the National Alcohol and Drug Use Research in Brazil show that, about 2 million Brazilians have smoked crack at least once at some point in their lives, ranking the country as the largest crack consumer in the whole world [2]. Thus, crack cocaine use is considered a serious public health problem in many places around the world, for example, Brazil, since psychosomatic injury, social exclusion, and high morbidity and mortality rates can be observed [3].

Several systemic health conditions are associated with the use of psychoactive substances (a natural or synthetic chemical taken via any route capable of acting on the central nervous system altering an individual’s behavior), especially crack cocaine (a smokable form of cocaine), with the cardiovascular, respiratory, neurological and gastrointestinal being some of the target systems [4]. In this sense, it is coherent to assume that the oral mucosa is a possible target, since the main form of crack consumption is through inhalation. The supporting mechanism to this assumption has its basis in some local effects, such as smoke heat, harmful effects of the chemical content of the drug, tissue necrosis due to gingival friction, vasoconstriction and a lower salivary flow rate [5]. Thus, cellular harmful outcomes as a result of developing oral lesions could be related to the abuse of such substances [6,7].

Even though several studies have approached the body effect of high consumption of crack cocaine [8,9,10], there is still minimal knowledge of its harmful effects on oral mucosal cells at the cellular and molecular level. In line with this limitation, this systematic review aimed to answer the following question: Are cytomorphogenetic events correlated with oral mucosal lesions induced by crack cocaine use?

## 2. Materials and Methods

This systematic review was carried out in accordance with the Preferred Reporting Items for Systematic Reviews and Meta-Analyses (PRISMA) statement 2020 criteria. The PICOS strategy (P (oral mucosa), I (crack cocaine exposure), C (control group), O (genotoxicity), S (clinical studies)) was used as a guide.

### 2.1. Inclusion Criteria

Published papers were included in our systematic review if they met the following criteria: (1) studies investigating genetic damage in vivo; (2) studies published in English; (3) studies that provided data with clear scientific standards. Through our scientific search strategy, we decided to focus on some techniques used for evaluating potential alterations in the oral mucosa (at different levels), such as micronucleus, cytomorphometry, AgNOR techniques and oral clinical inspection.

### 2.2. Exclusion Criteria

Published papers were excluded if they met the following criteria: (1) conference abstracts, reviews, editorials and letters; (2) full text not available in English; (3) unavailable data/unextractable data; (4) multigenerational studies; (5) incomplete or unclear results; (6) in vitro; (7) in vivo not conducted in humans.

### 2.3. Databases and Search Strategy

Electronic searches were undertaken in January 2023 in PubMed, SCOPUS and Web of Science electronic databases without regard to publication date or geographic region with the following keywords and Boolean operators: (“Crack” OR “Crack cocaine”) AND (“Genotoxicity” OR “DNA damage” OR “genetic damage” OR “DNA breakage” OR “genetic injury” OR “DNA injury” “chromosome damage” OR “oral mucosal lesion” “cell proliferation”). In addition, manual search of articles and cited/related articles was performed to identify publications that might have been missed by the primary database searches. The search approach was applied consistently across all databases.

Duplicates were removed upon identification. After duplicate removal, full texts were read to confirm eligibility by two authors (TGP and DAR). Disagreements were resolved between the two reviewers through discussion when consensus was reached.

### 2.4. Data Extraction and Quality Assessment

Two reviewers (TGP and DAR) independently extracted information from the included studies. The following data were extracted from the articles: authors, year and country of study, organs or cell types, time of exposure, assay, number of cells evaluated, genotoxicity assay used, blind, statistical analysis, negative control and main results. Data were tabulated and analyzed descriptively in Microsoft Office Excel 2019 (Microsoft^®^ software, version 16, Redmond, WA, USA).

The quality assessment of chosen studies was assessed independently by two reviewers (TGP and DAR). For this purpose, all variables (confounders) were considered in the study. If the study did not control one variable (confounder), it was considered STRONG (Low Risk of Bias) at the final rating. If the study did not control two variables, it was considered MODERATE (Medium Risk of Bias) at the final rating. Finally, studies that did not control three or more variables were categorized as WEAK (Low Risk of Bias) at the final rating, as described elsewhere [11].

## 3. Results

### 3.1. Study Selection

**Figure 1:**
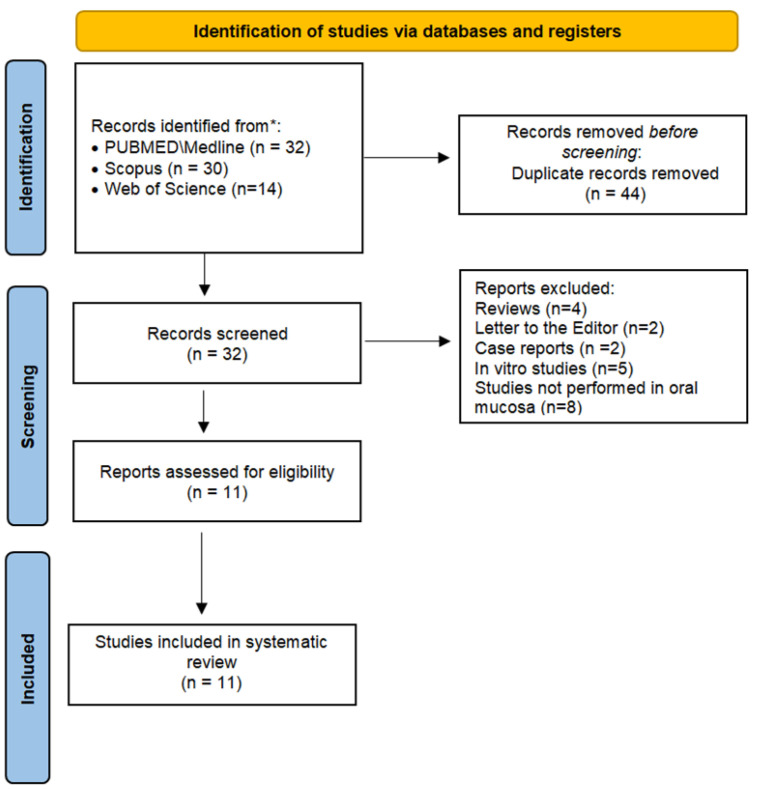
Flowchart of the study investigating the cytomorphogenetic events in oral mucosa from crack cocaine users.

Figure 1 depicts a flowchart of the current systematic review’s study selection process. A total of 76 references were retrieved in the initial search. Full manuscripts from 11 articles composed the analysis performed in this systematic review.

### 3.2. Study Characteristics

The included articles were published between 1994 and 2020 and were conducted in Brazil (n = 8), Germany (n = 2) and the USA (n = 1). Also, it is important to highlight that some studies only conducted oral mucosa clinical analysis (oral lesions), whilst others focused on a cellular/molecular-level evaluation.

### 3.3. Variables Related to Crack Use, Cytomorphogenetic Changes and Oral Lesions

Table 1 describes the main characteristics of studies that analyzed variables related to crack cocaine exposure at the cellular and molecular levels. In total, 492 individuals were evaluated in the crack cocaine group and 472 in the control (non-exposed) group. From the seven studies that evaluated the cellular and/or molecular alterations in cells, four studies used the MN test and three evaluated AgNORS. The number of counted cells evaluated in these studies varied from 50 to 1000. Table 2 shows the main characteristics of the four studies that evaluated oral mucosal lesions induced by crack cocaine. The total number of participants addicted included in the four studies was 185. In 50% of the studies, the clinical evaluation was performed by a specialist in the oral medicine field.

### 3.4. Main Results

All studies (11 out of 11) showed that the abuse of this substance was associated with increased oral mucosa alterations at the cellular, molecular or clinical levels. In other words, clinical analysis for oral mucosal lesions revealed an increased prevalence of oral lesions [5,7,16,17], and cellular analysis showed positive results closely related to genotoxicity (micronucleus), proliferative activity (AgNor immunoexpression) and changes in the cytoplasmic–nucleus ratio [2,3,7,12,13,14,15]. Nonetheless, the scarcity of studies that establish a correlation between oral lesions and molecular and/or cellular changes does not allow for drawing conclusions about this potential relationship. Such findings are summarized in Table 3.

### 3.5. Quality Assessment

The quality assessment is described in Table 4. A total of six studies [1,2,3,6,15,16] were classified as strong, two studies [12,14] were considered as moderate and three were considered to be weak [7,13,17], according to the chosen criteria, with eight of the evaluated studies considered to be either strong or moderate.

## 4. Discussion

This study aimed to assess if, and to what level, cellular and/or molecular changes can predict oral mucosal lesions induced by crack cocaine use. For this purpose, some studies performed assays to verify the genotoxic outcomes [3,6,7,12,13,14]; two papers performed a cytomorphometric analysis [2,15], and four studies evaluated the presence of oral mucosa lesions in crack cocaine users [5,7,16,17].

Our results revealed that six articles were considered to be strong, while two were considered moderate, adding up to three studies considered as weak. Taken as a whole, we assume that most of the assessed studies had good quality for evaluating either cellular or molecular changes associated or not with clinical evaluation, confirming reliable results. To achieve these objectives, some confounders were identified in this systematic review as such assays require specific parameters to guarantee proper evaluation performances and, thus, reliable results. Many genotoxicity tests are used worldwide due to their simplicity, robustness and time- and cost-effectiveness in targeting mutagenicity and, for that, may lack academic rigidity at some level. These assays play a pivotal role in identifying possible carcinogenesis since they evaluate the initiation phase of chemical carcinogenesis.

The micronucleus assay is widely used worldwide and is considered a sensitive method to detect chromosomes or fragments inside the cytoplasm of eukaryotic cells. The results of this study demonstrated that there were four out of the six studies in which genotoxicity was assessed using the micronucleus assay, indicating positive genotoxicity in all four of them [7,12,13,14]. Regarding the number of cells evaluated in the micronucleus assays, all studies evaluated 1000 cells per slide, which was considered to be a confounder in the rating categorizations to properly assess studies’ quality [18]. It has been suggested that a minimum of 2000 differentiated cells for treatment should be scored per sample for a proper analysis [19].

As an increased NA/CA ratio is one of the major signs of premalignant and malignant lesions [20], such analysis is also widely used and, in this systematic review, all of the analyzed studies suggested positive morphological alterations (2 out of 2) [2,15]. Furthermore, it is known that AgNORs is abundant in cells with high proliferative activity. In this method, black-brown spots are counted and analyzed based on their distribution pattern in the nucleus by measuring the occupied area with a light microscope [3]. A total of three studies identified high proliferative activity in the oral mucosa cells of crack cocaine users [3,6,13]. Such results confirm the participation of crack cocaine in the promotion phase of carcinogenesis in the oral mucosa. Certainly, these cellular and molecular data could predict the incidence of oral lesions in crack cocaine users, especially in some places considered at high risk of oral cancer, such as the floor of the mouth and lateral border of tongue. In fact, all evaluated studies that conducted clinical analysis observed that crack/cocaine addiction was significantly associated with oral mucosa lesions [5,7,16,17]. According to Cury et al. [16], the most prevalent lesions found in crack cocaine users were traumatic ulcers and actinic cheilitis, followed by fistulae associated with a retained dental root. In a study conducted by Mitchell-Lewis et al. [17], the authors observed unusual lesions at the midline of the hard palate and candidiasis induced by crack cocaine. Antoniazzi et al. [7] suggested that the most prevalent lesions observed in oral mucosa lesions of crack cocaine users were spot/plaques followed by ulcer/fissures and papule/nodules, respectively. Also, this study suggested that oral lesions were 2.02-fold more frequent, and micronuclei were 3.54-fold more frequent in crack users, implying some association with clinical and cellular changes in the oral mucosa. However, the authors suggest that part of the impact of crack on the occurrence of lesions was influenced by tooth loss, gingivitis and dental trauma [7]. It is important to stress that the study conducted by Antoniazzi et al. [7] was the only one to evaluate both oral mucosal lesions and the incidence of micronucleus assay in buccal cells. However, these authors did not find potentially malignant disorders or oral cancer. Recognizing the significant efforts and the outstanding achievements for clarifying the incidence of oral mucosa lesions in crack cocaine users, these articles, however, were categorized as weak at final rating in the quality assessment. Particularly, a letter to the Editor was published on the basis of proper quality evaluation criteria, revealing that the manuscript did not control some important variables, such as DNA-specific stain as well as the establishment of exclusion criteria [21]. Therefore, such findings should be interpreted cautiously.

As some studies suggest that crack cocaine abusers also smoke tobacco (and even marijuana) and that their consumption (combined or not) may be harmful and induce clastogenic events in the oral mucosa as well, it is necessary to highlight that six studies formally informed that part of the experimental group smoked anything rather than crack tobacco, two studies informed that 100% also used tobacco and three studies failed to disclose or report it. Regarding alcohol use, six studies informed that part of the crack cocaine users also drank alcohol and, lastly, only three studies informed that crack cocaine users were also marijuana users (at different percentages) [12,14].

A total of five studies applied some exclusion criteria, such as patients with systemic diseases, malignant tumor history, orthodontic appliance users and medication users, among others [3,7,12,13,16]. Additionally, the description of blind analysis was seen in the methodology of six studies out of the eight, whilst the rest failed to inform on this. Finally, all the included studies adequately described the statistical test applied when performing the data analysis.

## 5. Conclusions

In summary, although the type of assay and sample selection criteria may have influenced the outcome, our results demonstrate that crack use may induce changes at the cellular and molecular level closely related to genotoxicity [22], proliferative activity and abnormal phenotype. Taking into account the high-vulnerability condition of this group of people, being continuously exposed to physical, chemical or biological agents, a correlation between such changes and oral mucosa lesions still needs further investigation and elucidation through other clinical studies in humans. This aspect is certainly a relevant limitation of the study. As a result, there is a need for well-structured clinical studies on this topic to elucidate if the molecular changes detected have some relation with the development of specific oral lesions or potentially oral malignant disorders. Moreover, experimental studies in rodents would be a good alternative to elucidate if, and to what extent, crack cocaine induces genotoxicity in mammalian cells.

## Figures and Tables

**Table 1:** Main characteristics of studies that evaluated the effect of crack cocaine in oral mucosa cells in cellular and molecular levels.

Author/Year	Site of Smear	N	Age	Technique	Number of Evaluated Cells	Stain	Evaluated Parameters	Blind Analysis	Proper Statistic Description	Negative Control
Rabelo et al. (2020) [3]	Cheek mucosa	D (crack and cocaine): 38 (all males) C: 121 (all males)	>18	AgNOR	100 cell/nuclei	Silver nitrate	% of cells with more than 1, 2, 3 or 4 AgNORs/nucleus	--	Yes	Yes
Antoniazzi et al. (2018) [7]	Cheek mucosa	D (crack): 106/27 (females); 79 (males) C: 106/27 (females); 79 (males)	D (crack): 25.0 C: 22.5	MN Assay	1000 cells	Giemsa	Count cell	Yes	Yes	Yes
Albini. et al. (2017) [2]	Floor of the mouth	D: 234 (all males) C: 120 (all males)	D: 35.5 ± 10.2 C: 36.1 ± 11.3	NA, CA, and NA/CA ratio	50 cells	Papanicolaou technique	Inflammation score (n° of areas) Presence of keratinization; enucleated superficial cells; MN; and binucleation	Yes	Yes	Yes
Webber et al. (2016) [12]	Border of the tongue and floor of the	D (crack): 26/24 (males); 2 (females) A: 29/17(males); 12 (females) C: 35 (17 males and 18 females)	D: 40.94 ± 11.02 A: 45.83 ± 9.89 C: 39.62 ± 12.79	MN Assay AgNOR	1000 cells 50 cell/nuclei	Feulgen Silver Nitrate	Count cell N° of AgNORS/nucleus	Yes	Yes	Yes
Oliveira et al. (2014) [13]	Cheek mucosa	D (crack and cocaine): 30 (gender not specified) C: 30 (males only)	D: 31.4 ± 9.3 C: 33.6 ± 11.6	MN Assay	1000 cells	Feulgen/Fast Green	Count Cell	--	Yes	Yes
Thiele et al. (2013) [6]	Oral mucosa	D (crack and cocaine): 30 (males only) C: 30 (males only)	21–51 yo (most are 21–31 yo)	AgNOR	100 cell/nuclei	Nitrate silver	AgNORS/nucleus	Yes	Yes	Yes
Almeida et al. (2012) [14]	Oral mucosa	D (crack): 10 C: 10	D: 15.5 ± 1.43 C: 20.2 ± 0.98 yo	MN Assay	1000 cells	Feulgen	Count cell	Yes	Yes	Yes
Woyceichoski et al. (2008) [15]	Oral mucosa	D (crack): 20 C: 20	D: 24.3 C: 27.1	NA, CA, and NA/CA ratio	50 cells	Papanicolaou technique	Nuclear, cytoplasmatic area and nucleus-to-cytoplasm area ratio	Yes	Yes	Yes

MN = Micronuclei; OML: oral mucosa lesions; -- = Not described; N/A = not applicable. D: Drug user; C: Controls: NA: Nuclear; CA; cytoplasmic.

**Table 2:** Main characteristics of studies investigating oral mucosal lesions induced by crack cocaine.

Author/Year	Site of Smear	Age	Conducted by a Specialized Professional	Technique	Number of Addicted Patients	Exclusion Criteria	Evaluated Parameters	Proper Statistic Description	Negative Control
Cury et al. (2018) [16]	Oral mucosa	>18	Yes	Clinical evaluation	40	Yes	Prevalence of oral mucosal lesions	Yes	Yes
Antoniazzi et al. (2018) [7]	Oral mucosa	D (crack): 25.0 C: 22.5	No	Clinical evaluation	106	Yes	Prevalence of oral mucosal lesions	Yes	Yes
Sordi et al. (2014) [1]	Oral mucosa and saliva	>18	Yes	Clinical evaluation	35	No	Salivary flow rate and prevalence of oral mucosal lesions	Yes	Yes
Mitchell-Lewis et al. (1994) [17]	Oral mucosa	D: 38–54	No	Clinical evaluation	4	No	Oral mucosal lesions features (size, clinical appearance and symptoms)	Yes	Yes

**Table 3:** Main findings of studies investigating the effects of crack cocaine in oral mucosa.

Authors/Year	Cellular and/or Molecular Findings	Clinical Findings
Rabelo et al. (2020) [3]	↑ Cell proliferation	-
Cury et al. (2018) [16]	-	↑ Oral mucosal lesions
Antoniazzi et al. (2018) [7]	↑ MN	↑ Oral mucosal lesions
Sordi et al. (2017) [5]	-	↑ Oral mucosal lesions
Albini. et al. (2017) [2]	↑ NA/CA ↑ Inflammation	-
Webber et al. (2016) [12]	↑ MN	-
Oliveira et al. (2014) [13]	↑ MN ↑ Cell proliferation	-
Thiele et al. (2013) [6]	↑ Cell proliferation	-
Almeida et al. (2012) [14]	↑ MN	-
Woyceichoski et al. (2008) [15]	↑ NA/CA	-
Mitchell-Lewis et al. (1994) [17]	-	↑ Oral mucosal lesions

↑ = Increased; MN: micronucleus; NA = nuclear area; CA = cytoplasmic area; - = not evaluated.

**Table 4:** Quality assessment and final rating of the studies.

Authors/Year	Number of Confounders	Details	Final Rating
Rabelo et al. (2020) [3]	1	Blind analysis	Strong
Cury et al. (2018) [16]	0	-	Strong
Antoniazzi et al. (2018) [7]	3	Stain; total number of cells evaluated and clinical evaluation not conducted by a professional	Weak
Sordi et al. (2017) [5]	1	Lack of exclusion criteria	Strong
Albini. et al. (2017) [2]	0	-	Strong
Webber et al. (2016) [12]	2	Number of volunteers and total number of cells evaluated	Moderate
Oliveira et al. (2014) [13]	3	Number of volunteers, blind analysis, and total number of cells evaluated	Weak
Thiele et al. (2013) [6]	1	Number of volunteers	Strong
Almeida et al. (2012) [14]	2	Number of volunteers and total number of cells evaluated	Moderate
Woyceichoski et al. (2008) [15]	1	Number of volunteers	Strong
Mitchell-Lewis et al. (1994) [17]	3	Number of volunteers; lack of exclusion criteria and clinical evaluation not conducted by a professional	Weak

## Data Availability

Data sharing is not available for this article.
